# Early-Life Exposure to Particulate Matter and Sleep in Chinese Toddlers: The Roles of Particle Size and Particulate Constituents

**DOI:** 10.3390/toxics14080653

**Published:** 2026-07-25

**Authors:** Shu Wu, Qian Xu, Weiyin Zhuang, Xiaonan Gu, Yujing Chen, Qian Chen, Yu Liu, Mengfan Li, Lizi Lin, Li Cai, Hongwei Tu

**Affiliations:** 1 Department of Maternal and Child Health, School of Public Health, Sun Yat-sen University, Guangzhou 510080, China; wush56@mail2.sysu.edu.cn (S.W.); xuqian36@mail2.sysu.edu.cn (Q.X.); zhuangwy6@mail2.sysu.edu.cn (W.Z.); guxn@mail2.sysu.edu.cn (X.G.); liuy2387@mail2.sysu.edu.cn (Y.L.); limf55@mail2.sysu.edu.cn (M.L.); linlz@mail.sysu.edu.cn (L.L.); 2 Guangdong Provincial Center for Disease Control and Prevention, Guangzhou 511400, China; 3 JC School of Public Health and Primary Care, The Chinese University of Hong Kong, Hong Kong SAR 999077, China; 1155209014@link.cuhk.edu.hk; 4 The Third Affiliated Hospital of Guangzhou Medical University, Guangzhou 510150, China; 2021683073@gzhmu.edu.cn

**Keywords:** the first 1000 days of life, PM_2.5_, chemical constituents, sleep, birth cohort, toddlers

## Abstract

The impact of particulate matter (PM) on pediatric sleep has been documented, yet the roles of particle size and particulate constituents remain poorly understood. This study aims to evaluate the associations between early-life exposure to different sizes of PM and specific chemical constituents of PM_2.5_ and sleep in toddlers. In this birth cohort in Guangzhou, China, 514 mother–child pairs with follow-ups at age two between 2017 and 2018 were eligible for participation. Using high-resolution ground-level grid data, we estimated PM exposure (PM_1_, PM_2.5_, PM_10_) and major chemical constituents of PM_2.5_ during the early-life period. The sleep of two-year-old toddlers was assessed using the Chinese version of the Brief Infant Sleep Questionnaire. We used multivariable linear and logistic regression models to analyze the associations between PM exposure and sleep. Quantile g-computation was applied to assess the joint exposure effects of PM_2.5_ chemical constituents on sleep. An interquartile range (IQR) increase in PM_1_, PM_2.5_, and PM_10_ exposure during early life was associated with reduced nighttime sleep duration (β = −0.18; 95%CI: −0.32, −0.04; β = −0.23; 95%CI: −0.38, −0.08; β = −0.21; 95%CI: −0.35, −0.07). Combined early-life exposure to PM_2.5_ constituents correlated with reduced nighttime sleep duration, with SO_4_^2−^ being a major contributor. Regardless of particle size and PM_2.5_ constituents, we identified consistent findings during pregnancy. We found positive associations between prenatal PM exposure and reduced nighttime sleep in two-year-old toddlers. The PM_2.5_ constituent SO_4_^2−^ appears to be the major contributor to these associations.

## 1. Introduction

Sleep disruptions have become a significant public health issue, affecting many children [1]. Sleep is essential for the growth and maturation of the central nervous system in young children [2]. A study in China involving 1129 infants aged 1 to 23 months found that 66% experienced sleep disturbances, with 34% having recurring nighttime awakenings and 33% having difficulty initiating sleep [3]. Insufficient or low-quality sleep among infants is linked to impaired cognitive function, behavior issues, and emotional disorders [4]. Persistent sleep disorders into adulthood increase the risk of metabolic, cardiovascular, and respiratory diseases [5]. The first 1000 days of life, from conception to age two, are crucial for establishing sleep patterns. During this period, the nervous system forms a network for sleep regulation and develops a stable sleep–wake rhythm [6,7]. Therefore, it is essential to prevent sleep problems during this critical period.

Sleep quality is influenced by lifestyle, social interactions, psychological health, and environmental conditions [8,9]. Recent epidemiological studies have linked particulate matter (PM) pollution to sleep disturbances [10,11]. In 2021, China’s average PM_2.5_ and PM_10_ levels were 30 µg/m^3^ and 54 µg/m^3^, respectively, well above the WHO guidelines of 5 µg/m^3^ for PM_2.5_ and 15 µg/m^3^ for PM_10_ [12]. Smaller PM particles are more toxic because they penetrate deeper into the respiratory system [13]. PM_2.5_ is associated with adverse effects on the central nervous system through oxidative stress and neuroinflammation [14] and has been linked to changes in placental methylation in circadian pathway genes during the third trimester of pregnancy [15]. Research on the impact of PM_1_ exposure on sleep is limited due to scarce environmental monitoring data.

The chemical composition of PM, in addition to its size, significantly affects its toxicity [16]. Moreover, PM often acts as a carrier for various toxic and carcinogenic elements, such as heavy metals and polycyclic aromatic hydrocarbons, which are potentially important constituents that further compound its health risks [13]. A study in Beijing found prenatal exposure to PM_2.5_ or its constituents was linked to impaired fetal growth [17]. Similarly, a study in Boston showed long-term exposure to black carbon, a PM_2.5_ constituent, was associated with reduced sleep duration in adult males [18]. However, no studies have examined the impact of specific chemical constituents of PM_2.5_ on toddlers’ sleep. Most research has focused on older children, adolescents, and adults, leaving a gap regarding younger children.

Therefore, this study aims to investigate the roles of PM with varying particle size and the chemical constituents of PM_2.5_ on toddlers’ sleep during their first 1000 days, addressing this gap in the literature.

## 2. Materials and Methods

### 2.1. Participants

Participants for this research were selected from a Guangzhou-based birth cohort study, which was registered at the ClinicalTrials.gov registry (available at https://www.clinicaltrials.gov (accessed on 10 April 2026)) with the trial registration number NCT03023293 [19]; the registration date of this cohort study is 18 January 2017. The study involved pregnant women receiving routine prenatal check-ups at Yuexiu District Maternal and Child Health Care Hospital between March 2017 and November 2018. Inclusion criteria were a gestational age of 20 to 28 weeks and an age range of 20 to 45 years for the expectant mothers. Exclusion criteria included pre-existing medical conditions such as diabetes, cardiovascular disease, thyroid disorders, blood-related illnesses, polycystic ovary syndrome, infections during pregnancy, multiple pregnancies, and mental health disorders. The study focused on sleep outcomes of 514 toddlers at age two. The research protocol was approved by the ethics committee of the School of Public Health at Sun Yat-sen University (Approval No.: SYSU-PHME [2016] No. 014; Approval Date: 28 December 2016), and written informed consent was obtained from all participants.

We followed the Strengthening the Reporting of Observational Studies in Epidemiology (STROBE) reporting guideline for cohort studies. Data were analyzed from 20 January to 20 March 2024.

### 2.2. Particulate Matter Exposure Measurements

To determine particulate matter exposure, we geocoded participants’ residential locations and assigned gridded measurements of PM_1_, PM_2.5_, PM_10_, and the chemical constituents of PM_2.5_ to these locations. Daily concentration values were compiled to calculate average exposure levels over the initial 1000 days of life, from the last menstrual period to two years postnatal. The China High Air Pollutants (CHAP) dataset, with a 10 km × 10 km resolution, provided data on PM_1_ and PM_10_ using a space-time extremely randomized trees (STET) model [20,21], which integrates ground-based measurements, satellite remote sensing, atmospheric reanalysis, and model simulations [22]. The STET model effectively derives daily PM_1_ and PM_10_ concentrations with cross-validation coefficients of 0.77 and 0.82, respectively [20,21]. Data for PM_2.5_ and its chemical constituents were sourced from the Tracking Air Pollution in China (TAP) framework, which also operates at a 10 km × 10 km resolution. The daily concentration data for PM and its chemical constituents were directly downloaded from the reported CHAP and TAP datasets, rather than estimated independently by our team for the study period. The TAP dataset uses a random tree model incorporating ground observations, satellite-retrieved aerosol optical depth, and refined land use parameters [23]. It provides estimates for the five major chemical constituents of PM_2.5_ in China: sulfate (SO_4_^2−^), nitrate (NO_3_^−^), ammonium (NH_4_^+^), organic matter (OM), and black carbon (BC) [24]. These five specific constituents were selected for our analysis because they represent the predominant chemical components contributing to the total mass of ambient PM_2.5_ in China, and they possess robust, validated high-resolution exposure estimates within the TAP framework [23,24].

### 2.3. Sleep Outcomes Measurements

At age two, we assessed children’s sleep patterns using the Chinese version of the Brief Infant Sleep Questionnaire (BISQ), following the guidelines from the Chinese Sleep Hygiene Guidelines for children aged 0 to 5 years [25]. The BISQ is a validated tool for identifying developmental sleep patterns in infants, confirmed through comparison with actigraphy and sleep diaries [26]. Parents reported their child’s sleep habits over the past month, including bedtime routines, wake-up times, daytime nap duration, sleep onset latency (SOL), frequency of nighttime awakenings, experiences of nightmares, and perceived sleep regularity. Inadequate sleep was defined as less than 11 h of total sleep per day. Difficulty initiating sleep was indicated by a SOL of more than 30 min, and frequent nighttime awakenings were defined as occurring three or more nights per week. Parents’ perceptions of irregular sleep patterns and nightmares measured sleep quality disruptions. Nighttime sleep duration was calculated from bedtime, SOL, and wake-up time, while total sleep duration included both nighttime and daytime sleep.

### 2.4. Covariates

During the initial phase of the study, we conducted face-to-face interviews to collect sociodemographic details about the mothers, including age, education, household income, and smoking or secondhand smoke exposure. Information on gravidity, birthdates, and gender of the children was retrieved from the hospital’s birth registry. We also collected data on factors potentially affecting toddlers’ sleep through parental questionnaires, which included the duration of exclusive breastfeeding, sleep habits, ability to fall asleep independently, and secondhand smoke exposure at home.

Prior epidemiological evidence has also documented significant associations between sleep patterns and exposure to other ambient pollutants, including nitrogen dioxide (NO_2_) and ozone (O_3_) [27,28]. Therefore, we estimated daily concentrations of NO_2_ and O_3_ at a 10 km resolution using the CHAP dataset [29,30]. The normalized difference vegetation index (NDVI), derived from MODIS satellite imagery, assessed residential greenness levels. We calculated the mean levels of NO_2_, O_3_, and NDVI over the first 1000 days of life, including pregnancy and up to two years postpartum.

### 2.5. Statistical Analysis

We summarized the demographic features and sleep metrics of the participants using descriptive statistics, such as means with standard deviations or percentage distributions. To assess the impact of demographic variables and toddler gender on sleep outcomes, we used *t*-tests or one-way ANOVA for continuous data and the chi-square test for nominal data.

We further analyzed the data using multivariable linear regression to assess associations between particulate matter exposure, the chemical composition of PM_2.5_, and sleep durations (total, night, and day). Logistic regression models calculated odds ratios (ORs) for various sleep quality measures, including sleep insufficiency, trouble initiating sleep, irregular sleep patterns, frequent night waking, and nightmares. The joint effect of the five primary chemical constituents of PM_2.5_ was examined using the quantile g-computation technique [31]. Specifically, we utilized quartiles (four quantiles) for this analysis. The cut-off points were defined based on the continuous concentration distributions of each specific constituent and were determined separately for each distinct exposure period (during pregnancy, 0–2 years postpartum, and the first 1000 days of life). Our regression models controlled for a comprehensive set of potential confounders, as outlined in the directed acyclic graph in Figure S1. These included maternal age, work status, education, family income, pregnancy history, the toddlers’ sex, duration of exclusive breastfeeding, sleep practices, passive smoke exposure, and environmental factors such as NO_2_ and O_3_ levels, as well as residential greenness.

To verify the consistency of our results, we conducted additional sensitivity analyses. First, we removed children born prematurely or with low birth weight (*n* = 26). Second, we controlled for particulate matter exposure during the postnatal period as a covariate for prenatal exposure, and vice versa, over the first two years of life.

All statistical analyses were performed using R version 4.1.2 (https://www.R-project.org, (accessed on 10 April 2026)), with statistical significance set at *p* < 0.05 for two-tailed tests.

## 3. Results

### 3.1. Demographic Characteristics and Sleep Outcomes

Table 1 presents an overview of 514 mother–child pairs in the study, with 50.97% male toddlers and 17.51% advanced maternal age (≥35 years old). The comparisons of maternal demographic characteristics between the baseline and follow-up populations are detailed in Supplementary Table S1. Bed-sharing was reported in 91.60% of cases. Parents reported that 82 (15.95%) toddlers had difficulty falling asleep, 81 (15.76%) experienced frequent nighttime awakenings, and 74 (14.40%) had irregular sleep patterns. On average, two-year-old toddlers slept 12.18 ± 1.13 h per day, including 10.06 ± 0.97 h at night.

### 3.2. Concentrations of Inhalable Particles

**Figure 1:**
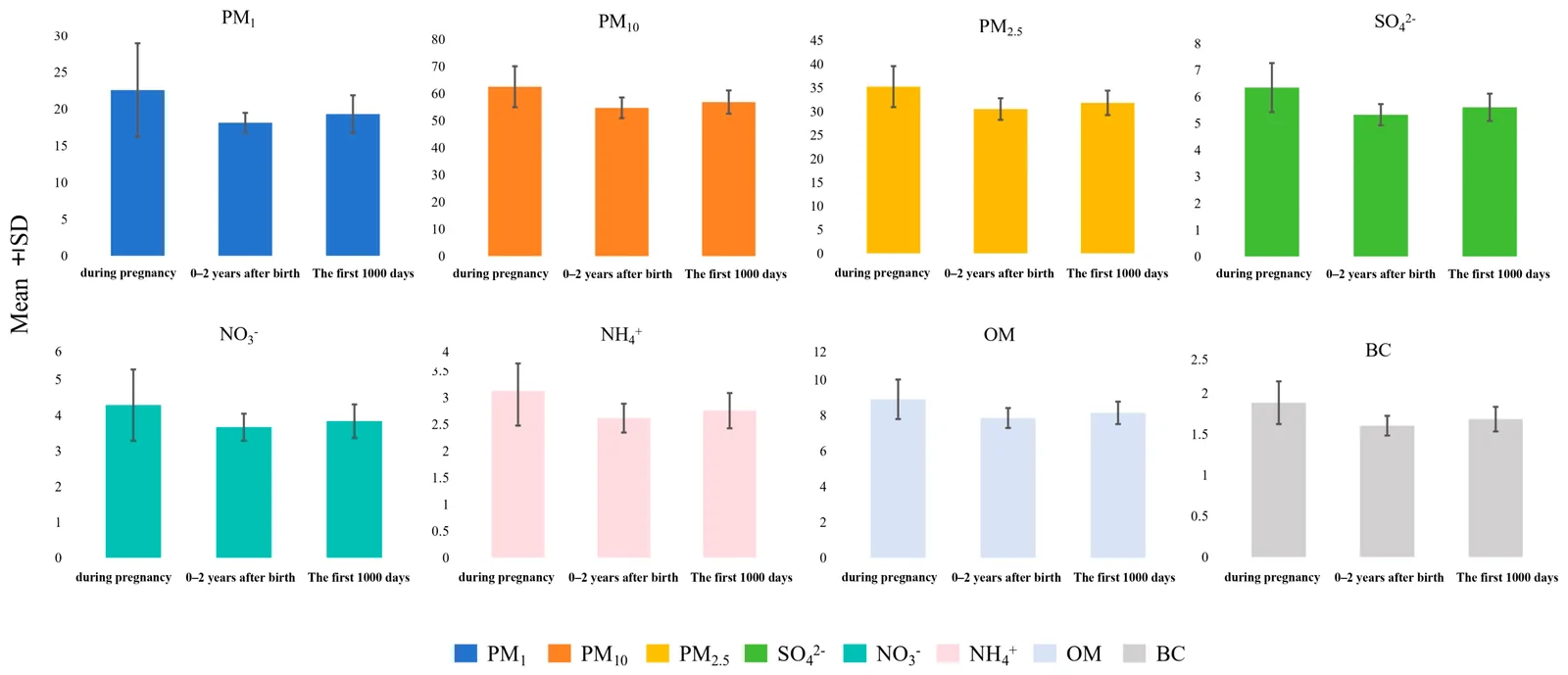
Concentrations of particulate matter and PM_2.5_ constituents in early life (N = 514, unit: μg/m^3^). Abbreviations: SD, standard deviation; PM_1_, particulate matter with an aerodynamic diameter ≤ 1 μm; PM_10_, particulate matter with an aerodynamic diameter ≤ 10 μm; PM_2.5_, particulate matter with an aerodynamic diameter ≤ 2.5 μm; SO_4_^2−^, sulfate; NO_3_^−^, nitrate; NH_4_^+^, ammonium; OM, organic matter; BC, black carbon.

Figure 1 displays the concentrations of particulate matter and PM_2.5_ constituents. Average daily concentrations of PM_1_, PM_2.5_, and PM_10_ during the first 1000 days of life were 19.29 ± 2.56 μg/m^3^, 31.78 ± 2.58 μg/m^3^, and 56.79 ± 4.28 μg/m^3^, respectively. Among PM_2.5_ constituents, organic matter showed the highest concentration at 8.13 ± 0.63 μg/m^3^, followed by SO_4_^2−^ at 5.60 ± 0.51 μg/m^3^.The pairwise Spearman correlation matrix between ambient PM_2.5_ and its composition during the first 1000 days is presented in Supplementary Figure S2.

### 3.3. Associations Between Exposure to Particulate Matter and Sleep Outcomes

Table 2 in our study outlines the correlations between particulate matter exposure and sleep quality in two-year-old children. Notably, we found generally consistent associations throughout the initial 1000 days of life, spanning both prenatal and postnatal periods up to age two. We found that higher PM_2.5_ exposure was linked to a 129% increased likelihood of parent-reported irregular sleep (adjusted odds ratio [OR], 2.29; 95% confidence interval [CI], 1.16, 4.49). Increases in PM_1_, PM_2.5_, and PM_10_ levels were associated with shorter nighttime sleep durations in toddlers, with β coefficients of −0.18 (95% CI: −0.32, −0.04), −0.23 (95% CI: −0.38, −0.08), and −0.21 (95% CI: −0.35, −0.07), respectively.

Regarding the chemical constituents of PM_2.5_ (Supplementary Table S2), we observed higher odds of irregular sleep with ORs of 1.82 (95% CI: 1.07, 3.02) for SO_4_^2−^, 1.88 (95% CI: 1.11, 3.11) for NO_3_^−^, 1.83 (95% CI: 1.11, 2.91) for NH_4_^+^, 1.87 (95% CI: 1.06, 3.24) for OM, and 1.90 (95% CI: 1.06, 3.36) for BC. Moreover, increases in SO_4_^2−^, NO_3_^−^, NH_4_^+^, OM, and BC levels were associated with shorter nighttime sleep durations, with β coefficients of −0.16 (95% CI: −0.29, −0.02) for SO_4_^2−^, −0.15 (95% CI: −0.28, −0.02) for NO_3_^−^, −0.14 (95% CI: −0.27, −0.01) for NH_4_^+^, −0.18 (95% CI: −0.31, −0.05) for OM, and −0.19 (95% CI: −0.33, −0.06) for BC.

Table 3 demonstrates the collective influence of the five PM_2.5_ constituents on sleep using the qg-computation model. The combined effect of these constituents was associated with a reduction in nighttime sleep duration and higher risks of irregular sleep in two-year-old children during both the first 1000 days and during pregnancy (β = −0.11, 95% CI: −0.19, −0.03; β = −0.10, 95% CI: −0.18, −0.01; OR= 1.53, 95% CI: 1.18, 1.98; OR= 1.42, 95% CI: 1.10, 1.84). The weight index indicated that SO_4_^2−^ significantly contributed to the adverse effects across most associations.

### 3.4. Sensitivity Analysis

The associations between particulate matter exposure, PM_2.5_ chemical constituents, and sleep outcomes remained consistent in the sample excluding children with preterm birth or low birth weight (Tables S3 and S4). Additionally, the associations for prenatal exposure persisted even after adjusting for postnatal exposure (Supplementary Table S5), and the effects of postnatal exposure also remained consistent when adjusting for prenatal exposure (Supplementary Table S6). Furthermore, the qg-computation models for the association of mixture exposures with additional sleep quality measures (difficulty falling asleep, nocturnal awakenings, and nightmares) are provided in Supplementary Table S7.

## 4. Discussion

This study explored how exposure to different sizes of PM and specific PM_2.5_ chemical constituents during the first 1000 days of life relates to sleep outcomes in two-year-old toddlers. We identified positive correlations between prenatal PM exposure and decreased nighttime sleep in two-year-old toddlers, irrespective of particle size. The PM_2.5_ constituent SO_4_^2−^ seems to be the primary contributor to these associations. This study is the first prospective birth cohort to examine the relationship between early-life PM exposure and sleep problems in two-year-old toddlers, taking into account different particle sizes and PM_2.5_ constituents.

Previous research on the association between PM and children’s sleep showed inconsistent findings. A cross-sectional study in China with 59,754 children reported associations between PM_1_, PM_2.5_, and PM_10_ and increased sleep disorders [32]. Another cohort in Mexico indicated that PM_2.5_ exposure during different gestation periods affected sleep quality differently in 4–5-year-olds [11]. However, a cross-sectional study in Egypt found no significant associations between PM_10_ concentrations and overall sleep disturbance (*p* = 0.073) [10], which might be due to the heterogeneity of the sleep assessment period and methods. Our study revealed negative associations between PM_1_, PM_2.5_, and PM_10_ exposure during the first 1000 days of life and nighttime sleep duration in two-year-old toddlers. Coarse PM like PM_10_ can cause inflammation in the upper respiratory tract and disrupt ventilation-perfusion coupling [33], potentially leading to sleep apnea and reduced sleep quality [34]. For toddlers in their first 1000 days, our findings also showed a positive association between PM_2.5_ exposure and parent-perceived irregular sleep. These correlations may be explained through the following mechanisms. The sleep–wake cycle is regulated by brainstem functions, a process that relies on the equilibrium of neurotransmitters [35]. PM_2.5_ exposure has been proven to be related to changes in serotonin levels, which is an important neurotransmitter regulating sleep patterns [36]. Evidence also suggests that PM can indirectly affect sleep regulation levels by inducing brain inflammation [37,38]. Although direct evidence linking PM_1_ exposure to sleep disturbances is currently lacking, its potential impact cannot be ignored, given its ability to penetrate deep into the lungs and potentially affect cellular and circulatory systems [39], with possible implications for neurodegenerative diseases linked to disrupted sleep patterns [40,41]. Future research should delve deeper into the biological pathways connecting PM_1_ exposure to toddler sleep, essential for monitoring urban PM_1_ concentrations and pollution management.

Our study is the first to investigate the associations between PM_2.5_ chemical constituents and toddlers’ sleep. Elevated BC concentrations during pregnancy, the first two years postpartum, and the first 1000 days of life correlated with an increased risk of sleep irregularities and shorter nighttime sleep duration in toddlers (Table S2). A study in Boston found that prolonged BC exposure reduced sleep duration in adult males [18], and a retrospective cohort study showed a higher risk of poor sleep quality in university students exposed to BC [42]. BC, a significant part of PM_2.5_, mainly comes from the incomplete combustion of fossil fuels and biomass [43]. It consists of fractal chain-like aggregates of small carbon spheres (10–50 nm in diameter) with the highest light-absorbing capacity among PM_2.5_ constituents [44]. Animal and ex vivo human placental models have shown that PM_2.5_ constituents like BC can cross the placental barrier, affecting placental function and fetal health [45]. Paul et al. [46] found in a mouse model that PM constituents in the placenta reduced its efficiency, linking this to impaired lung development in offspring. Bové et al. [47] observed PM_2.5_ constituents on the fetal side of the human placenta, indicating that BC migrates from maternal circulation to various placental sites. The accumulation of BC in the placenta correlates with maternal BC exposure during pregnancy, suggesting a dose–response relationship [47]. Future studies should conduct comprehensive toxicological evaluations of PM_2.5_ constituents and clarify their metabolic pathways in placental tissue to understand their impact on placental function and offspring health.

Our results showed that mixed exposure to PM_2.5_ chemical constituents during pregnancy and the first 1000 days of life was linked to an increased risk of irregular sleep and decreased nighttime sleep duration in toddlers. This finding aligns with results from single-constituent exposure. Among the five chemical constituents, SO_4_^2−^ was the second most prevalent and played a primary role in the combined exposure effect. While no studies have specifically examined the relationship between PM_2.5_ constituents and toddlers’ sleep, previous research has found prenatal SO_4_^2−^ exposure to be associated with impaired fetal growth and higher BMI Z-scores in early childhood [17]. The mechanisms by which SO_4_^2−^ affects toddlers’ sleep remain unclear, but prior studies suggest PM, SO_4_^2−^, and NO_3_^−^ accelerate amyloid protein aggregation linked to Alzheimer’s disease [48]. Since toddler sleep is closely tied to nervous system development [49], further research is needed to explore how PM_2.5_ constituents impact sleep biologically. SO_4_^2−^ primarily forms from sulfur dioxide released by fossil fuel combustion, while NO_3_^−^ comes from nitrogen oxides emitted by motor vehicles [50]. The NO_3_^−^ to SO_4_^2−^ ratio is an important metric for assessing mobile and stationary pollution sources, and a ratio exceeding 1 indicates a higher proportion of mobile source PM_2.5_ [51]. During our study, the NO_3_^−^/SO_4_^2−^ ratio was 0.68, indicating that fixed sources like factories and fossil fuel combustion were the main contributors to PM_2.5_. In the Guangzhou area, SO4^2−^ is heavily influenced by industrial emissions and coal combustion from surrounding manufacturing hubs. Although regional clean air policies have driven a downward trend in overall particulate mass in recent years, secondary inorganic aerosols like SO4^2−^ remain prominent due to complex atmospheric photochemical reactions [52]. Additional measures should be taken to reduce SO_4_^2−^ pollution sources in Guangzhou.

This is the first study to investigate links between PM_2.5_ chemical constituent exposure during the first 1000 days of life and toddlers’ sleep patterns. However, our research has certain limitations. First, traffic-related noise and the indoor environment are important factors affecting sleep. Our study did not collect data on traffic and indoor environments, which might introduce some limitations to the interpretation of our results. Nonetheless, we have controlled for other environmental factors, such as residential greenness and NO_2_, to minimize potential confounding effects. Second, parent-reported questionnaires might overestimate sleep duration and underestimate sleep problems [53], but the Brief Infant Sleep Questionnaire we employed has been validated against objective sleep actigraphy [26]. Finally, this single-center study was conducted exclusively in a Southern Chinese metropolitan area, necessitating future validation through multicenter investigations with geographically diverse cohorts.

## 5. Conclusions

We found positive associations between prenatal PM exposure and reduced nighttime sleep in two-year-old toddlers, regardless of particle size. The PM_2.5_ constituent SO_4_^2−^ appears to be the major contributor to these associations. These findings should be interpreted in light of certain limitations, notably the lack of objective data on indoor air quality and traffic noise, the reliance on parent-reported sleep questionnaires rather than actigraphy, and the single-center nature of the cohort. Considering the high prevalence of sleep disorders in early childhood and their potential to persist into later life, policies to control PM pollution should address the diverse chemical constituents and their impact on toddler sleep health. For expectant mothers and parents, we recommend taking proactive preventive measures, such as utilizing indoor air purifiers and minimizing outdoor activities during periods of severe PM pollution, to help safeguard toddlers against potential sleep disruptions.

## Figures and Tables

**Table 1:** General characteristics of study population stratified by child’s sex (N = 514).

		Child’s sex	
Characteristics	All Participants	Male (*n* = 262)	Female (*n* = 252)
**Maternal characteristic at pregnancy**			
Maternal age, *n* (%)			
≥35 years	90 (17.51)	54 (20.61)	36 (14.29)
<35 years	424 (82.49)	208 (79.39)	216 (85.71)
Maternal employment ^a^, *n* (%)			
No	133 (27.19)	70 (28.34)	63 (26.03)
Yes	356 (72.81)	177 (71.66)	179 (83.97)
Maternal education level ^a^, *n* (%)			
Senior high school and below	164 (32.80)	74 (29.13)	90 (36.59)
Junior college	158 (31.60)	89 (35.04)	69 (28.05)
University and above	178 (35.60)	91 (35.83)	87 (35.36)
Monthly household income ^a^, *n* (%)			
<6000 RMB	214 (42.88)	100 (39.22)	114 (46.72)
6000~10,000 RMB	170 (34.07)	88 (34.51)	82 (33.61)
>10,000 RMB	115 (23.05)	67 (26.27)	48 (19.67)
Maternal smoking and/or passive smoking ^a^, *n* (%)			
No	365 (72.42)	187 (72.48)	178 (72.36)
Yes	139 (27.58)	71 (27.52)	68 (27.64)
**Child’s characteristics by age 2 years**			
Low birthweight			
No	504 (98.05)	257 (98.09)	247 (98.02)
Yes	10 (1.95)	5 (1.91)	5 (1.98)
Exclusive breastfeeding, *n* (%)			
<6 months	339 (65.95)	173 (66.03)	166 (65.87)
≥6 months	175 (34.05)	89 (33.97)	86 (34.13)
Sleep location, *n* (%)			
Falling asleep in bed alone	43 (8.37)	20 (7.63)	23 (9.13)
Bed-sharing with family members	471 (91.63)	242 (92.37)	229 (90.87)
Sleep quality, *n* (%)			
Insufficient sleep ^b^	95 (18.48)	47 (17.94)	48 (19.05)
Difficulty falling asleep ^c^	82 (15.95)	35 (13.36)	47 (18.65)
Frequent nighttime awakenings ^d^	81 (15.76)	44 (16.79)	37 (14.68)
Parent-perceived irregular sleep	74 (14.40)	42 (16.03)	32 (12.70)
Nightmare	26 (5.06)	10 (3.81)	16 (6.35)
Sleep time (h), *mean* ± *SD*			
total sleep time	12.18 ± 1.13	12.20 ± 1.06	12.19 ± 1.20
sleep time at night	10.06 ± 0.97	10.00 ± 0.92	10.15 ± 1.01
sleep time during daytime	2.12 ± 0.67	2.20 ± 0.65	2.04 ± 0.69
**Environmental exposure during the first 1000 days of life**			
NO_2_, μg/m^3^, mean ± SD	50.04 ± 4.10	50.04 ± 4.34	50.01 ± 3.88
O_3_, μg/m^3^, mean ± SD	94.22 ± 4.83	94.21 ± 4.83	94.27 ± 4.87
NDVI, mean ± SD	0.33 ± 0.09	0.33 ± 0.10	0.33 ± 0.09

Abbreviations: SD, standardized deviation; NO_2_, nitrogen dioxide; O_3_, ozone; NDVI, normalized difference vegetation index. ^a^, Maternal employment had missing data for 4.9%, maternal education level for 2.7%, Monthly household income for 2.9%, and maternal smoking for 1.9%. ^b^, Insufficient sleep: total sleep duration < 11 h. ^c^, Difficulty falling asleep: sleep onset latency > 30 min. ^d^, Frequent nighttime awakenings ≥ 3 nights/week. Data were presented as mean ± SD or *n* (%).

**Table 2:** Associations between exposure to particulate matter and sleep in toddlers.

Sleep	Difficulty to Fall Asleep	Irregular Sleep	Nocturnal Awaking	Nightmare	Total Sleep Duration	Nighttime Sleep Duration	Daytime Sleep Duration
	*OR (95% CI)*	*OR (95% CI)*	*OR (95% CI)*	*OR (95% CI)*	*β ^a^ (95% CI)*	*β (95% CI)*	*β (95% CI)*
The first 1000 days							
PM_1_	1.05 (0.59, 1.81)	0.95 (0.49, 1.78)	0.88 (0.47, 1.58)	1.34 (0.49, 3.39)	−0.09 (−0.25, 0.07)	**−0.18** **(−0.32, −0.04)**	0.09 (−0.00, 0.18)
PM_2.5_	1.11 (0.60, 2.01)	**2.29** **(1.16, 4.49)**	1.09 (0.56, 2.04)	0.69 (0.18, 2.14)	−0.11 (−0.28, 0.06)	**−0.23** **(−0.38, −0.08)**	**0.12** **(0.02, 0.22)**
PM_10_	0.95 (0.47, 1.99)	0.98 (0.44, 2.26)	1.43 (0.67, 3.19)	2.00 (0.56, 7.89)	−0.10 (−0.26, 0.07)	**−0.21** **(−0.35, −0.07)**	**0.12** **(0.02, 0.21)**
During pregnancy							
PM_1_	0.70 (0.42, 1.14)	1.46 (0.85, 2.51)	0.90 (0.54, 1.49)	1.27 (0.55, 2.86)	−0.09 (−0.27, 0.08)	**−0.19** **(−0.34, −0.04)**	0.09 (−0.01, 0.20)
PM_2.5_	0.93 (0.57, 1.50)	**1.89** **(1.10, 3.23)**	0.89 (0.52, 1.48)	0.82 (0.32, 1.89)	−0.07 (−0.23, 0.08)	**−0.17** **(−0.30, −0.03)**	**0.10** **(0.01, 0.19)**
PM_10_	0.79 (0.41, 1.52)	1.27 (0.62, 2.66)	0.96 (0.49, 1.90)	1.49 (0.48, 4.80)	−0.04 (−0.22, 0.13)	**−0.17** **(−0.31, −0.02)**	**0.12** **(0.02, 0.22)**
0~2 years old after birth							
PM_1_	1.07 (0.80, 1.42)	0.73 (0.53, 1.01)	0.94 (0.70, 1.26)	1.27 (0.77, 2.09)	−0.05 (−0.15, 0.05)	**−0.09** **(−0.18, −0.00)**	0.04 (−0.02, 0.10)
PM_2.5_	0.84 (0.44, 1.58)	**1.99** **(1.01, 3.86)**	0.93 (0.47, 1.80)	0.89 (0.27, 2.58)	−0.12 (−0.29, 0.06)	**−0.23** **(−0.38, −0.08)**	**0.11** **(0.01, 0.21)**
PM_10_	0.71 (0.37, 1.38)	1.05 (0.51, 2.20)	1.23 (0.62, 2.49)	2.11 (0.68, 7.00)	−0.12 (−0.29, 0.05)	**−0.21** **(−0.36, −0.07)**	0.09 (−0.01, 0.19)

Abbreviation: OR, odds ratio; CI, confidence interval; PM_1_, particulate matter with an aerodynamic diameter ≤ 1 μm; PM_2.5_, particulate matter with an aerodynamic diameter ≤ 2.5 μm; PM_10_, particulate matter with an aerodynamic diameter ≤ 10 μm; ^*a*^ Estimated effects associated with a per interquartile range increase in exposures to PM_1_, PM_2.5_ and PM_10_. The main regression model was adjusted for maternal age, maternal employment, maternal education, monthly household income, gravidity, child’s sex, duration of exclusive breastfeeding, sleeping habits of toddlers, secondhand smoke, NO_2_, O_3_, and residential greenness. Bold values indicate statistical significance (*p* < 0.05).

**Table 3:** Qg-computation models for the association of **mixture exposures (five PM_2.5_ constituents)** with sleep (N = 514).

Sleep Quality	Joint Effect ^a^		Index Weight				
	[*OR*/*β (95% CI)*] ^b^	*p*	SO_4_^2−^	NO_3_^−^	NH_4_^+^	OM	BC
**The first 1000 days**							
irregular sleep	**1.53 (1.18, 1.98)**	**0.001**	0.794	−0.487	0.206	−0.280	−0.233
nighttime sleep duration	**−0.11 (−0.19, −0.03)**	**0.009**	0.728	0.207	−0.703	0.065	0.297
daytime sleep duration	0.05 (−0.10, 0.01)	0.100	−0.394	−0.205	0.658	−0.400	0.342
**during pregnancy**							
irregular sleep	**1.42 (1.10, 1.84)**	**0.007**	0.445	−0.425	0.555	−0.128	−0.446
nighttime sleep duration	**−0.10 (−0.18, −0.01)**	**0.027**	−0.318	−0.259	−0.344	−0.006	−0.074
daytime sleep duration	**0.06 (0.00, 0.11)**	**0.048**	−0.049	−0.455	0.315	−0.496	0.685
**0–2 years** **after birth**							
irregular sleep	**1.29 (1.01, 1.64)**	**0.044**	−0.020	0.289	−0.980	−0.443	0.711
nighttime sleep duration	−0.06 (−0.15, 0.02)	0.159	1.000	−0.320	−0.301	−0.258	−0.120
daytime sleep duration	0.05 (−0.01, 0.11)	0.084	−0.816	−0.184	0.564	0.155	0.281

Abbreviation: OR, odds ratio; CI, confidential interval; SO_4_^2−^, sulfate; NO_3_^−^, nitrate; NH_4_^+^, ammonium; OM, organic matter; BC, black carbon. ^a^ Estimated effects associated with a per interquartile range increase in exposures to five PM_2.5_ constituents. ^b^ Odds Ratio (OR) was used to describe irregular sleep, and β was used to describe nighttime and daytime sleep duration. The main regression model was adjusted for maternal age, maternal employment, maternal education, monthly household income, gravidity, child’s sex, duration of exclusive breastfeeding, sleeping habits of toddlers, secondhand smoke, NO_2_, O_3_, and residential greenness. Bold values indicate statistical significance (*p* < 0.05).

## Data Availability

The TAP dataset is available at http://tapdata.org.cn/ (accessed on 10 April 2026), and the CHAP dataset is available at https://weijing-rs.github.io/product.html, (accessed on 10 April 2026).

## Supplementary Materials

The following supporting information can be downloaded at: https://www.mdpi.com/article/10.3390/toxics14080653/s1, Table S1. The comparisons of maternal demographic characteristics between baseline and follow-up populations; Table S2. Associations between exposure to chemical composition of PM_2.5_ and sleep in toddlers; Table S3. Sensitivity analysis of the association between particulate matter and sleep in 2-year-olds excluded preterm infants and low-birth-weight infants; Table S4. Sensitivity analysis of the association between chemical composition of PM_2.5_ and sleep in 2-year-olds excluded preterm and low-birth-weight infants; Table S5. Sensitivity analysis of the association between prenatal exposure and sleep in 2-year-olds adjusted for postnatal exposure; Table S6. Sensitivity analysis of the association between postnatal exposure and sleep in 2-year-olds adjusted for prenatal exposure; Table S7. Qg-computation models for the association of mixture exposures (five PM_2.5_ components) with sleep (N = 514); Figure S1. Directed acyclic graph for the hypothesized causal relationship between particulate matter and PM_2.5_ components and toddlers’ sleep; Figure S2. Pairwise Spearman correlation matrix between ambient PM_2.5_ and its composition during the first 1000 days (N = 514).

## Abbreviations

The following abbreviations are used in this manuscript:

PM	Particulate matter
SO_4_^2−^	Sulfate
NO_3_^−^	Nitrate
NH_4_^+^	Ammonium
OM	Organic matter
BC	Black carbon
BISQ	Brief Infant Sleep Questionnaire
CHAP	China High Air Pollutants
STET	Space-time extremely randomized trees
TAP	Tracking Air Pollution in China
NO_2_	Dioxide
O_3_	Ozone
NDVI	Normalized difference vegetation index
SOL	Sleep onset latency
IQR	Interquartile range
OR	Odds ratio
CI	Confidence interval
SD	Standard deviation
