# Supplementary material for: Early-Life Exposure to Particulate Matter and Sleep in Chinese Toddlers: The Roles of Particle Size and Particulate Constituents
Source: Toxics. 2026 Jul 25;14(8):653. doi: 10.3390/toxics14080653 (PMC13517296; doi:10.3390/toxics14080653)
Supplement: Supplementary file 1 [file toxics-14-00653-s001.zip › toxics-4407963-supplementary.pdf]

Early-life exposure to particulate matter and sleep in Chinese toddlers: the roles of particle size and particulate constituents

Appendix 1

Table S1. The comparisons of maternal demographic characteristics between baseline and follow-up populations.

Table S2. Associations between exposure to chemical composition of PM2.5 and sleep in toddlers.

Table S3. Sensitivity analysis of the association between particulate matter and sleep in 2-year-olds excluded preterm infants and low-birth-weight infants.

Table S4. Sensitivity analysis of the association between chemical composition of PM2.5 and sleep in 2-year-olds excluded preterm and low-birth-weight infants.

Table S5. Sensitivity analysis of the association between prenatal exposure and sleep in 2-year-olds adjusted for postnatal exposure.

Table S6. Sensitivity analysis of the association between postnatal exposure and sleep in 2-year-olds adjusted for prenatal exposure.

Table S7. Qg-computation models for the association of mixture exposures (five PM2.5 components) with sleep (N=514).

Figure S1. Directed acyclic graph for the hypothesized causal relationship between particulate matter and PM2.5 components and toddlers' sleep.

Figure S2. Pairwise Spearman correlation matrix between ambient PM2.5 and its composition during the first 1,000 days (N=514).

Table S1. The comparisons of maternal demographic characteristics between baseline and follow-up populations.

	Baseline (N=1029)	Follow-up (N=514)	*p* value
Maternal age, *n (%)*			0.486
≥35 years	171 (16.62)	90 (17.51)	
<35 years	858 (83.38)	424 (82.50)	

Maternal employment, *n (%)*			0.976
No	270 (26.24)	133 (27.19)	
Yes	759 (73.76)	356 (72.81)	
Maternal education level, *n* (%)			0.318
Senior high school and below	411 (39.94)	164 (32.80)	
Junior college	306 (29.74)	158 (31.60)	
University and above	312 (30.32)	178 (35.60)	
Monthly household income, *n (%)*			0.936
<6,000 RMB	461 (44.80)	214 (42.88)	
6,000~10,000 RMB	260 (25.27)	170 (34.07)	
>10,000 RMB	308 (29.93)	115 (23.05)	

Data were presented as n (%).

P value from chi-square test.

Table S2. Associations between exposure to chemical composition of $PM_{2.5}$ and sleep in toddlers.

Sleep	Difficulty to fall asleep	Irregular sleep	Nocturnal awaking	Nightmare	Total sleep duration	Nighttime sleep duration	Daytime sleep duration
	OR (95%CI)	OR (95%CI)	OR (95%CI)	OR (95%CI)	β [a]（95% CI)	β（95% CI)	β（95% CI)
The first 1000 days							
SO_4^{2-}	1.25 (0.79, 1.96)	1.82 (1.07, 3.02)	0.90(0.50, 1.50)	0.86 (0.28, 2.09)	-0.06 (-0.22, 0.10)	-0.16 (-0.29, -0.02)	0.09 (0.00, 0.19)
NO_3^-	1.28 (0.80, 2.01)	1.88 (1.11, 3.11)	0.68(0.34, 1.23)	0.76 (0.21, 1.92)	-0.07 (-0.22, 0.08)	-0.15 (-0.28, -0.02)	0.08 (-0.01, 0.16)
NH_4^+	1.27 (0.81, 1.93)	1.83 (1.11, 2.91)	0.77(0.40, 1.32)	0.73 (0.20, 1.80)	-0.07 (-0.22, 0.08)	-0.14 (-0.27, -0.01)	0.07 (-0.02, 0.16)
OM	1.17 (0.71, 1.88)	1.87 (1.06, 3.24)	0.92(0.52, 1.57)	0.83 (0.28, 2.07)	-0.07 (-0.23, 0.08)	-0.18 (-0.31, -0.05)	0.10 (0.02, 0.19)
BC	1.23 (0.73, 2.02)	1.90 (1.06, 3.36)	0.92(0.50, 1.61)	0.88 (0.28, 2.28)	-0.08 (-0.24, 0.08)	-0.19 (-0.33, -0.06)	0.11 (0.02, 0.20)

During pregnancy							
SO_4^{2-}	1.11 (0.74, 1.62)	1.68 (1.09, 2.55)	0.93 (0.59, 1.41)	0.97 (0.45, 1.89)	-0.06 (-0.20, 0.08)	-0.15 (-0.27, -0.03)	0.09 (0.01, 0.17)
NO_3^-	0.94 (0.52, 1.64)	1.88 (1.01, 3.45)	0.60 (0.31, 1.11)	0.81 (0.27, 2.13)	-0.07 (-0.24, 0.10)	-0.14 (-0.29, -0.01)	0.07 (-0.03, 0.17)
NH_4^+	0.97 (0.57, 1.61)	1.83 (1.05, 3.15)	0.67 (0.36, 1.20)	0.80 (0.28, 1.95)	-0.07 (-0.23, 0.10)	-0.14 (-0.28, -0.00)	0.07 (-0.02, 0.17)
OM	1.03 (0.65, 1.62)	1.75 (1.05, 2.89)	0.82 (0.49, 1.34)	0.83 (0.35, 1.85)	-0.05 (-0.20, 0.10)	-0.14 (-0.27, -0.01)	0.09 (0.00, 0.18)
BC	1.06 (0.73, 1.51)	1.60 (1.07, 2.39)	0.90 (0.60, 1.34)	0.94 (0.47, 1.76)	-0.05 (-0.18, 0.07)	-0.14 (-0.24, -0.03)	0.08 (0.01, 0.16)
0~2 years old after birth							
SO_4^{2-}	1.30 (0.80, 2.07)	2.09 (1.19, 3.63)	0.79 (0.43, 1.38)	1.05 (0.38, 2.45)	-0.04 (-0.20, 0.11)	-0.12 (-0.25, 0.01)	0.08 (-0.01, 0.17)
NO_3^-	1.31 (0.79, 2.14)	2.14 (1.23, 3.69)	0.82 (0.42, 1.48)	1.11 (0.38, 2.51)	-0.06 (-0.22, 0.10)	-0.13 (-0.26, 0.01)	0.07 (-0.03, 0.16)
NH_4^+	1.35 (0.85, 2.10)	2.10 (1.26, 3.48)	0.87 (0.45, 1.50)	1.03 (0.35, 2.25)	-0.06 (-0.21, 0.10)	-0.11 (-0.25, 0.02)	0.06 (-0.03, 0.15)

OM	1.24 (0.67, 2.25)	2.43 (1.22, 4.88)	0.86 (0.43, 1.65)	1.16 (0.38, 3.21)	-0.09 (-0.25, 0.08)	-0.18 (-0.33, -0.04)	0.10 (0.00, 0.20)
BC	1.33 (0.72, 2.42)	2.45 (1.21, 4.97)	0.79 (0.39, 1.54)	1.22 (0.39, 3.43)	-0.09 (-0.26, 0.08)	-0.19 (-0.33, -0.05)	0.10 (0.00, 0.20)

Abbreviation: OR, odds ratio; CI, confidential interval; SO_4^{2-}, sulfate; NO_3^-, nitrate; NH_4^+, ammonium; OM, organic matter; BC, black carbon.

[a] Estimated effects associated with a per interquartile range increase in exposures to SO_4^{2-}, NO_3^-, NH_4^+, OM, and BC.

The main regression model was adjusted for maternal age, maternal employment, maternal education, monthly household income, gravidity, child's sex, duration of exclusive breastfeeding, sleeping habits of toddlers, secondhand smoke, NO_2, O_3, and residential greenness.

Table S3. Sensitivity analysis of the association between particulate matter and sleep in 2-year-olds, excluding preterm infants and low-birth-weight infants.

Sleep	Difficulty to fall asleep	Irregular sleep	Nocturnal awaking	Nightmare	Total sleep duration	Nighttime sleep duration	Daytime sleep duration
	OR (95%CI)	OR (95%CI)	OR (95%CI)	OR (95%CI)	β [a]（95% CI)	β（95% CI)	β（95% CI)
The first 1000 days							

PM_1	1.05 (0.59, 1.84)	0.91 (0.47, 1.71)	0.93 (0.49, 1.68)	1.34 (0.48, 3.46)	-0.05 (-0.22, 0.11)	-0.15 (-0.29, -0.01)	0.10 (0.00, 0.19)
$PM_{2.5}$	1.17 (0.63, 2.14)	2.16 (1.09, 4.23)	1.18 (0.60, 2.24)	0.61 (0.14, 2.03)	-0.07 (-0.24, 0.11)	-0.20 (-0.35, -0.05)	0.13 (0.03, 0.24)
PM_{10}	0.99 (0.48, 2.11)	0.95 (0.43, 2.16)	1.43 (0.67, 3.22)	1.59 (0.46, 6.23)	-0.07 (-0.24, 0.09)	-0.19 (-0.33, -0.05)	0.11 (0.02, 0.21)
During pregnancy							
PM_1	0.75 (0.44, 1.23)	1.36 (0.78, 2.34)	0.89 (0.52, 1.48)	1.23 (0.52, 2.87)	-0.05 (-0.23, 0.13)	-0.15 (-0.31, -0.00)	0.10 (0.00, 0.21)
$PM_{2.5}$	1.03 (0.62, 1.69)	1.77 (1.03, 3.05)	0.87 (0.50, 1.47)	0.66 (0.23, 1.65)	-0.04 (-0.19, 0.12)	-0.14 (-0.28, -0.01)	0.11 (0.01, 0.20)
PM_{10}	0.88 (0.44, 1.76)	1.22 (0.59, 2.57)	0.91 (0.45, 1.84)	1.08 (0.33, 3.68)	-0.03 (-0.21, 0.14)	-0.15 (-0.30, -0.00)	0.12 (0.02, 0.23)
0~2 years old after birth							
PM_1	1.04 (0.76, 1.42)	1.43 (0.67, 3.19)	0.98 (0.72, 1.33)	1.32 (0.79, 2.19)	-0.05 (-0.15, 0.06)	-0.08 (-0.17, 0.00)	0.04 (-0.02, 0.10)
$PM_{2.5}$	1.29 (0.62, 2.68)	3.07 (1.38, 6.97)	1.24 (0.58, 2.66)	0.92 (0.24, 3.07)	-0.08 (-0.26, 0.10)	-0.20 (-0.35, -0.05)	0.12 (0.02, 0.23)

PM_{10}	0.74 (0.35, 1.58)	0.97 (0.45, 2.13)	1.34 (0.65, 2.87)	2.50 (0.74, 9.20)	-0.10 (-0.27, 0.08)	-0.19 (-0.34, -0.04)	0.09 (-0.01, 0.19)

Abbreviation: OR, odds ratio; CI, confidence interval; PM_1, particulate matter with an aerodynamic diameter ≤1 μm; $PM_{2.5}$, particulate matter with an aerodynamic diameter ≤2.5 μm; PM_{10}, particulate matter with an aerodynamic diameter ≤10 μm.

[a] Estimated effects associated with a per interquartile range increase in exposures to PM_1, $PM_{2.5}$ and PM_{10}.

We excluded preterm and low-birth-weight infants (N=26). The main regression model was adjusted for maternal age, maternal employment, maternal education, monthly household income, gravidity, child's sex, duration of exclusive breastfeeding, sleeping habits of toddlers, secondhand smoke, NO_2, O_3, and residential greenness.

Table S4. Sensitivity analysis of the association between chemical composition of $PM_{2.5}$ and sleep in 2-year-olds, excluding preterm and low-birth-weight infants.

Sleep	Difficulty to fall asleep	Irregular sleep	Nocturnal awaking	Nightmare	Total sleep duration	Nighttime sleep duration	Daytime sleep duration
	OR (95%CI)	OR (95%CI)	OR (95%CI)	OR (95%CI)	β [a]（95% CI)	β（95% CI)	β（95% CI)
The first 1000 days							
SO_4^{2-}	1.31 (0.82, 2.07)	1.74 (1.02,	0.95 (0.53,	0.82 (0.24,	-0.03 (-0.19,	-0.14 (-0.27, 0.00)	0.11 (0.01, 0.20)

		2.89)	1.60)	2.11)	0.13)		
NO_3^-	1.34 (0.83, 2.12)	1.83 (1.07, 3.02)	0.73 (0.36, 1.32)	0.64 (0.15, 1.84)	-0.03 (-0.18, 0.12)	-0.12 (-0.25, 0.01)	0.09 (-0.00, 0.18)
NH_4^+	1.31 (0.84, 2.01)	1.76 (1.07, 2.82)	0.82 (0.42, 1.41)	0.64 (0.14, 1.79)	-0.03 (-0.18, 0.13)	-0.11 (-0.24, 0.02)	0.08 (-0.01, 0.17)
OM	1.23 (0.75, 2.00)	1.79 (1.02, 3.10)	0.99 (0.55, 1.70)	0.75 (0.23, 2.00)	-0.04 (-0.20, 0.11)	-0.16 (-0.29, -0.02)	0.12 (0.03, 0.21)
BC	1.30 (0.77, 2.16)	1.81 (1.00, 3.19)	0.98 (0.53, 1.73)	0.83 (0.24, 2.26)	-0.05 (-0.21, 0.11)	-0.17 (-0.31, -0.03)	0.12 (0.03, 0.21)
During pregnancy							
SO_4^{2-}	1.19 (0.79, 1.76)	1.58 (1.02, 2.40)	0.89 (0.56, 1.38)	0.86 (0.36, 1.79)	-0.04 (-0.18, 0.11)	-0.13 (-0.25, -0.01)	0.09 (0.01, 0.18)
NO_3^-	1.09 (0.60, 1.93)	1.78 (0.95, 3.29)	0.57 (0.29, 1.09)	0.63 (0.19, 1.87)	-0.03 (-0.20, 0.14)	-0.11 (-0.26, 0.04)	0.08 (-0.02, 0.19)
NH_4^+	1.09 (0.64, 1.82)	1.73 (1.03, 2.98)	0.65 (0.34, 1.18)	0.65 (0.20, 1.78)	-0.03 (-0.20, 0.14)	-0.11 (-0.26, 0.03)	0.08 (-0.01, 0.18)
OM	1.15 (0.71, 1.83)	1.66 (1.05, 2.76)	0.81 (0.47, 1.34)	0.66 (0.25, 1.60)	-0.02 (-0.18, 0.13)	-0.13 (-0.26, 0.01)	0.10 (0.01, 0.19)

BC	1.14 (0.78, 1.65)	1.52 (1.01, 2.27)	0.88 (0.58, 1.32)	0.82 (0.37, 1.63)	-0.03 (-0.16, 0.10)	-0.12 (-0.23, -0.01)	0.09 (0.02, 0.17)
0~2 years old after birth							
SO_4^{2-}	1.38 (0.85, 2.23)	2.06 (1.17, 3.57)	0.89 (0.48, 1.56)	1.03 (0.36, 2.44)	-0.01 (-0.17, 0.14)	-0.10 (-0.23, 0.03)	0.09 (-0.00, 0.18)
NO_3^-	1.37 (0.83, 2.25)	2.10 (1.20, 3.62)	0.92 (0.47, 1.65)	1.12 (0.37, 2.57)	-0.02 (-0.19, 0.14)	-0.10 (-0.24, 0.04)	0.08 (-0.02, 0.17)
NH_4^+	1.40 (0.87, 2.19)	2.06 (1.23, 3.40)	0.96 (0.51, 1.66)	1.04 (0.34, 2.33)	-0.02 (-0.18, 0.14)	-0.09 (-0.22, 0.05)	0.07 (-0.02, 0.16)
OM	1.33 (0.72, 2.46)	2.39 (1.19, 4.81)	1.00 (0.50, 1.95)	1.11 (0.35, 3.13)	-0.05 (-0.22, 0.12)	-0.16 (-0.31, -0.01)	0.11 (0.01, 0.21)
BC	1.43 (0.77, 2.64)	2.39 (1.18, 4.86)	0.92 (0.45, 1.81)	1.17 (0.35, 3.35)	-0.05 (-0.22, 0.11)	-0.17 (-0.31, -0.02)	0.11 (0.01, 0.21)

Abbreviation: OR, odds ratio; CI, confidential interval; SO_4^{2-}, sulfate; NO_3^-, nitrate; NH_4^+, ammonium; OM, organic matter; BC, black carbon.

[a] Estimated effects associated with a per interquartile range increase in exposures to SO_4^{2-}, NO_3^-, NH_4^+, OM, and BC. We excluded preterm and low-birth-weight infants (N=26). The main regression model was adjusted for maternal age, maternal employment, maternal education, monthly household income, gravidity, child's sex, duration of exclusive breastfeeding, toddlers' sleeping habits, secondhand smoke, NO_2, O_3, and residential greenness.

Table S5. Sensitivity analysis of the association between prenatal exposure and sleep in 2-year-olds adjusted for postnatal exposure.

Sleep	Difficulty to fall asleep	Irregular sleep	Nocturnal awaking	Nightmare	Total sleep duration	Nighttime sleep duration	Daytime sleep duration
	OR (95%CI)	OR (95%CI)	OR (95%CI)	OR (95%CI)	β [a]（95% CI)	β（95% CI)	β（95% CI)
PM_1	0.55 (0.19, 1.49)	1.79 (0.56, 5.86)	0.99 (0.81, 1.20)	1.47 (0.21, 9.80)	0.08 (-0.43, 0.60)	0.19 (-0.25, 0.63)	-0.10 (-0.39, 0.19)
PM_{10}	1.02 (0.58, 1.78)	1.78 (1.01, 3.15)	0.73 (0.42, 1.24)	0.76 (0.31, 1.82)	-0.02 (-0.33, 0.28)	-0.05 (-0.32, 0.21)	0.03 (-0.14, 0.21)
$PM_{2.5}$	1.09 (0.61, 1.94)	1.82 (1.01, 3.31)	0.72 (0.41, 1.26)	0.76 (0.29, 1.93)	-0.15 (-0.46, 0.16)	-0.18 (-0.45, 0.08)	0.04 (-0.14, 0.21)
SO_4^{2-}	1.62 (0.87, 3.03)	2.06 (1.12, 3.83)	0.63 (0.34, 1.15)	0.86 (0.29, 2.38)	-0.12 (-0.43, 0.20)	-0.17 (-0.44, 0.10)	0.06 (-0.12, 0.24)
NO_3^-	1.02 (0.60, 1.76)	1.58 (1.06, 2.40)	0.61 (0.36, 1.04)	0.80 (0.33, 1.96)	-0.15 (-0.46, 0.15)	-0.16 (-0.42, 0.11)	0.01 (-0.17, 0.18)
NH_4^+	1.06 (0.61, 1.86)	2.15 (1.12, 4.27)	0.60 (0.34, 1.04)	0.77 (0.30, 1.98)	-0.13 (-0.45, 0.18)	-0.15 (-0.41, 0.12)	0.02 (-0.16, 0.19)

OM	1.17 (0.68, 2.01)	1.77 (1.03, 3.08)	0.69 (0.40, 1.17)	0.80 (0.32, 1.91)	-0.12 (-0.41, 0.17)	-0.16 (-0.40, 0.09)	0.05 (-0.12, 0.21)
BC	1.33 (0.80, 2.23)	1.80 (1.08, 3.04)	0.68 (0.41, 1.13)	0.84 (0.35, 1.94)	-0.12 (-0.38, 0.15)	-0.16 (-0.39, 0.06)	0.05 (-0.10, 0.21)

Abbreviation: OR, odds ratio; CI, confidence interval; PM_1, particulate matter with an aerodynamic diameter ≤1 μm; $PM_{2.5}$, particulate matter with an aerodynamic diameter ≤2.5 μm; PM_{10}, particulate matter with an aerodynamic diameter ≤10 μm; SO_4^{2-}, sulfate; NO_3^-, nitrate; NH_4^+, ammonium; OM, organic matter; BC, black carbon.

[a] Estimated effects associated with a per interquartile range increase in exposures to PM_1, $PM_{2.5}$, PM_{10}, SO_4^{2-}, NO_3^-, NH_4^+, OM, and BC.

The main regression model was adjusted for maternal age, maternal employment, maternal education, monthly household income, gravidity, child's sex, duration of exclusive breastfeeding, toddlers' sleeping habits, secondhand smoke, NO_2, O_3, residential greenness and particulate matter exposure (PM_1, $PM_{2.5}$ and PM_{10}).0~2 years after birth.

Table S6. Sensitivity analysis of the association between postnatal exposure and sleep in 2-year-olds adjusted for prenatal exposure.

Sleep	Difficulty to fall asleep	Irregular sleep	Nocturnal awaking	Nightmare	Total sleep duration	Nighttime sleep duration	Daytime sleep duration
	OR (95%CI)	OR (95%CI)	OR (95%CI)	OR (95%CI)	β [a]（95% CI)	β（95% CI)	β（95% CI)
PM_1	1.16 (0.88, 1.55)	0.74 (0.53,	1.10 (0.73,	0.91 (0.42,	-0.10 (-0.27,	-0.10 (-0.25, 0.04)	0.01 (-0.09, 0.11)

		1.01)	1.68)	1.91)	0.08)		
PM_{10}	1.14 (0.99, 1.34)	1.06 (0.91, 1.27)	1.37 (1.16, 1.68)	1.26 (0.35, 5.46)	-0.07 (-0.38, 0.24)	-0.05 (-0.31, 0.22)	-0.02 (-0.20,0.16)
$PM_{2.5}$	1.18 (0.90, 1.57)	1.17 (0.86, 1.63)	1.68 (1.24, 2.39)	0.31 (0.05, 2.12)	0.10 (-0.36, 0.57)	-0.13 (-0.52, 0.27)	0.23 (-0.03, 0.50)
SO_4^{2-}	2.56 (0.43, 5.42)	1.64 (0.65, 3.93)	0.81(0.29, 2.06)	0.39 (0.06, 2.22)	0.38 (-0.00, 0.76)	0.20 (-0.12, 0.53)	0.18 (-0.04, 0.40)
NO_3^-	2.36 (0.60, 9.42)	2.10 (0.89, 4.97)	1.08 (0.45, 2.50)	0.55 (0.11, 2.34)	0.18 (-0.17, 0.53)	0.13 (-0.16, 0.43)	0.05 (-0.15, 0.25)
NH_4^+	3.09 (0.50, 8.71)	1.72 (0.77, 3.79)	1.07 (0.46, 2.33)	0.45 (0.08, 2.05)	0.17 (-0.16, 0.50)	0.13 (-0.15, 0.41)	0.04 (-0.15, 0.23)
OM	2.58 (0.69, 7.17)	3.16 (0.97, 6.50)	1.62 (0.50, 5.21)	0.42 (0.06, 2.99)	0.34 (-0.14, 0.81)	0.06 (-0.34, 0.47)	0.28 (0.00, 0.55)
BC	1.26 (0.93, 1.72)	2.82 (0.89, 9.03)	1.26 (0.39, 3.97)	0.46 (0.06, 3.37)	0.31 (-0.16, 0.79)	0.02 (-0.38, 0.42)	0.29 (0.02, 0.56)

Abbreviation: OR, odds ratio; CI, confidence interval; PM_1, particulate matter with an aerodynamic diameter ≤1 μm; $PM_{2.5}$, particulate matter with an aerodynamic diameter ≤2.5 μm; PM_{10}, particulate matter with an aerodynamic diameter ≤10 μm; SO_4^{2-}, sulfate; NO_3^-, nitrate; NH_4^+, ammonium; OM, organic matter; BC, black carbon.

[a] Estimated effects associated with a per interquartile range increase in exposures to PM_1, $PM_{2.5}$, PM_{10}, SO_4^{2-}, NO_3^-, NH_4^+, OM, and BC.

The main regression model was adjusted for maternal age, maternal employment, maternal education, monthly household income, gravidity, child's sex, duration of exclusive breastfeeding, toddlers' sleeping habits, secondhand smoke, NO_2, O_3, residential greenness and particulate matter exposure (PM_1, $PM_{2.5}$ and PM_{10}) during pregnancy.

Table S7. Qg-computation models for the association of mixture exposures (five $PM_{2.5}$ components) with sleep (N=514).

Sleep quality	Joint effect [a]		Index weight				
	[OR/β (95%CI)] [b]	p	SO_4^{2-}	NO_3^-	NH_4^+	OM	BC
The first 1000 days							
difficulty to fall asleep	0.87 (0.68, 1.09)	0.226	0.982	-0.103	0.018	-0.724	-0.173
nocturnal awaking	1.01 (0.80, 1.28)	0.911	0.873	0.127	-0.198	-0.338	-0.464
nightmare	0.88 (0.60, 1.28)	0.495	0.116	0.691	-0.724	-0.276	0.194
total sleep duration	-0.07 (-0.16, 0.03)	0.172	0.889	-0.043	-0.220	-0.736	0.111
during pregnancy							
difficulty to fall asleep	0.92 (0.73, 1.17)	0.505	-0.394	0.546	-0.562	0.454	0.044
nocturnal awaking	0.86 (0.68, 1.09)	0.211	0.208	-0.662	-0.338	0.241	0.551
nightmare	0.89 (0.60, 1.33)	0.577	-0.108	0.289	-0.461	-0.431	0.711
total sleep duration	-0.04 (-0.14, 0.06)	0.401	-0.170	-0.453	-0.218	-0.377	0.782
0-2 years after birth							
difficulty to fall asleep	0.83 (0.65, 1.06)	0.128	0.570	-0.242	0.430	-0.734	-0.024
nocturnal awaking	1.15 (0.91, 1.44)	0.238	0.356	-0.583	0.644	0.283	0.135

nightmare	0.99 (0.68, 1.44)	0.950	0.371	0.092	-0.166	-0.834	0.537
total sleep duration	-0.02 (-0.12, 0.09)	0.767	0.504	0.454	-0.979	0.042	-0.021

Abbreviation: OR, odds ratio; CI, confidential interval; SO_4^{2-}, sulfate; NO_3^-, nitrate; NH_4^+, ammonium; OM, organic matter; BC, black carbon.

[a] Estimated effects associated with a per interquartile range increase in exposures to five $PM_{2.5}$ constituents.

[b] Difficulty to fall asleep, nocturnal awaking and nightmares were described using Odds Ratio (OR), and total sleep duration was described using β.

The main regression model was adjusted for maternal age, maternal employment, maternal education, monthly household income, gravidity, child's sex, duration of exclusive breastfeeding, sleeping habits of toddlers, secondhand smoke, NO_2, O_3, and residential greenness.

Figure S1. Directed acyclic graph for the hypothesized causal relationship between particulate matter and $PM_{2.5}$ components and toddlers' sleep.

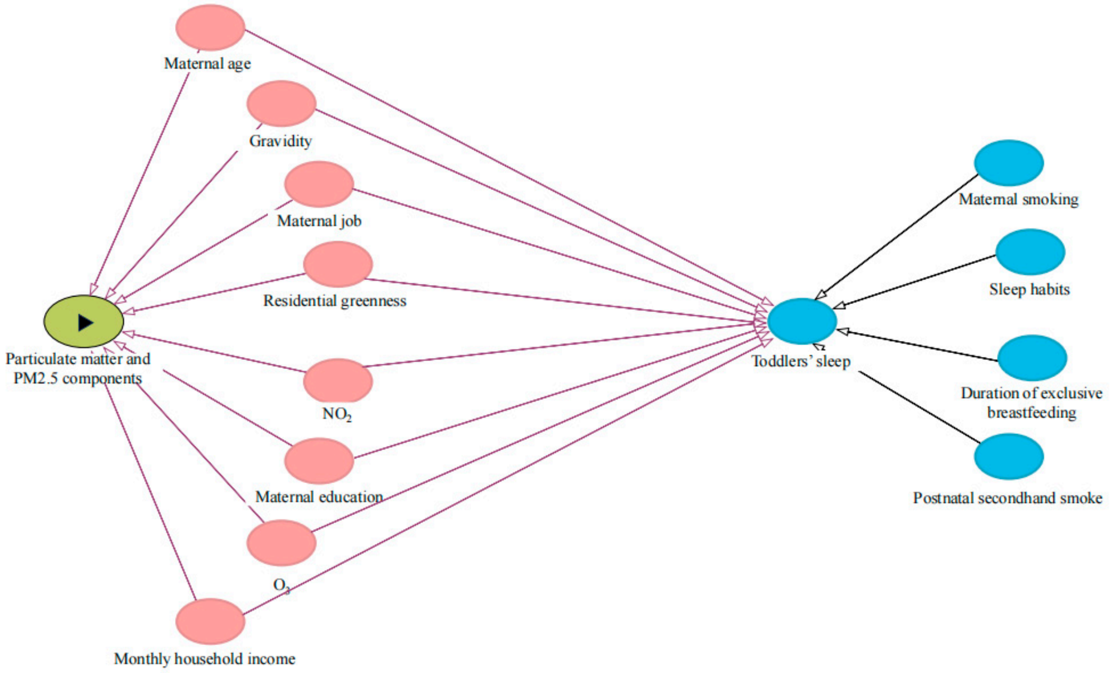

Abbreviations: $PM_{2.5}$, particulate matter with an aerodynamic diameter ≤2.5 µm.

Green circle: ancestor of exposure; Blue circle: ancestor of outcome; Red circle: ancestor of exposure and outcome; Red line: biasing path.

Graph was drawn using DAGitty version 3.1 (http://dagitty.net/).

Figure S2. Pairwise Spearman correlation matrix between ambient $PM_{2.5}$ and its composition during the first 1,000 days (N=514).

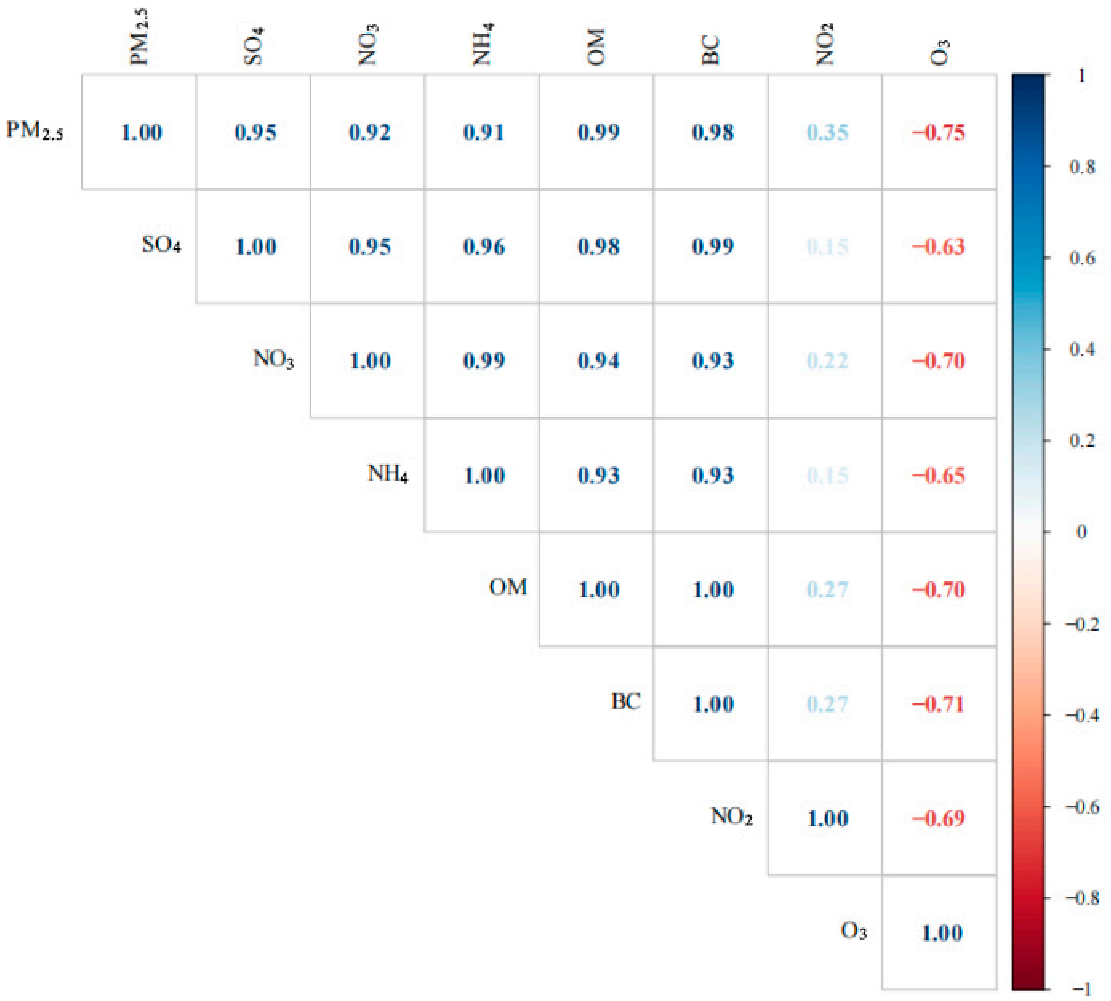

Abbreviations: $PM_{2.5}$, particulate matter with an aerodynamic diameter ≤2.5 µm (10 km resolution); SO_4^{2+}, sulfate; NO_3^-, nitrate; NH_4^+, ammonium; OM, organic matter; BC, black carbon; NO_2, nitrogen dioxide; O_3, ozone.
